# Distribution of *Scedosporium* species in soil from areas with high human population density and tourist popularity in six geographic regions in Thailand

**DOI:** 10.1371/journal.pone.0210942

**Published:** 2019-01-23

**Authors:** Natthanej Luplertlop, Watcharamat Muangkaew, Potjaman Pumeesat, San Suwanmanee, Pantira Singkum

**Affiliations:** Department of Microbiology and Immunology, Faculty of Tropical Medicine, Mahidol University, Bangkok Thailand; University of Innsbruck, AUSTRIA

## Abstract

*Scedosporium* is a genus comprising at least 10 species of airborne fungi (saprobes) that survive and grow on decaying organic matter. These fungi are found in high density in human-affected areas such as sewage-contaminated water, and five species, namely *Scedosporium apiospermum*, *S*. *boydii*, *S*. *aurantiacum*, *S*. *dehoogii*, and *S*. *minutisporum*, cause human infections. Thailand is a popular travel destination in the world, with many attractions present in densely populated areas; thus, large numbers of people may be exposed to pathogens present in these areas. We conducted a comprehensive survey of *Scedosporium* species in 350 soil samples obtained from 35 sites of high human population density and tourist popularity distributed over 23 provinces and six geographic regions of Thailand. Soil suspensions of each sample were inoculated on three plates of Scedo-Select III medium to isolate *Scedosporium* species. In total, 191 *Scedosporium* colonies were isolated from four provinces. The species were then identified using PCR and sequencing of the beta-tubulin (BT2) gene. Of the 191 isolates, 188 were *S*. *apiospermum*, one was *S*. *dehoogii*, and species of two could not be exactly identified. Genetic diversity analysis revealed high haplotype diversity of *S*. *apiospermum*. Soil is a major ecological niche for *Scedosporium* and may contain *S*. *apiospermum* populations with high genetic diversity. This study of *Scedosporium* distribution might encourage health care providers to consider *Scedosporium* infection in their patients.

## Introduction

*Scedosporium* is a genus of filamentous fungi with septate hyphae. These fungi are airborne saprobes that survive and grow on decaying organic matter, and thus, are frequently found in high density in human-affected areas such as sewage, contaminated water, and urban soil. The genus currently comprises 10 species, five of which, namely *Scedosporium apiospermum*, *S*. *boydii*, *S*. *aurantiacum*, *S*. *dehoogii*, and *S*. *minutisporum*, cause Scedosporiosis. [1]. This disease has recently emerged as a severe fungal infection from environmental sources. *Scedosporium* biology has been studied mainly after its isolation from the environment. In Thailand, *S*. *apiospermum* was reported in brain abscesses of near-drowning individuals [2]. Moreover, two Swiss tourists who nearly drowned in a tsunami disaster were infected with *S*. *apiospermum* [3].

The distribution of *Scedosporium* is of increasing clinical importance considering several severe *Scedosporium* species infections reported throughout Asia. In Japan, Nakamura *et al*. reported multiple brain abscesses caused by *S*. *aurantiacum* [4], and Shimizu *et al*. reported vertebral osteomyelitis caused by *S*. *apiospermum* in patients who nearly drowned during the Great East Japan Earthquake and Tsunami [5]. In Taiwan, Chen et al. [6] reported a case of disseminated *S*. *apiospermum* infection in a near-drowning patient. Thus, a unique feature of this environmental fungus is the infection of immunocompetent humans after near-drowning incidents. Therefore, clinicians and microbiologists should be aware of this possibility in regions with high endemicity. Furthermore, additional research on disease course and treatment is required.

Therefore, our team began to study *Scedosporium* species in their natural habitats in Thailand. In our initial study, we surveyed and isolated colonies of *Scedosporium* species in soil and water samples from 10 public parks in Bangkok, the capital and most populous city in Thailand. Three major *Scedosporium* species, namely *S*. *apiospermum*, *S*. *aurantiacum*, and *S*. *dehoogii*, were identified, but 16 sequences could not be identified, implying the possibility of additional *Scedosporium* species [7].

In this study, we conducted a more comprehensive survey of *Scedosporium* species in soil samples by investigating soil samples in Thailand from 35 public areas with high human population density and tourist popularity in six geographic regions of Thailand.

## Materials and methods

### Soil sample collection

Sampling at all locations in 35 public parks was authorized by the mayor’s office of the municipality or city in 23 provinces in six geographic regions: northern (Chiang Mai, Chiang Rai, and Nan Provinces), central (Phitsanulok, Nakhon Sawan, Nakhon Nayok, Phra Nakhon Si Ayutthaya, and Samut Songkhram Province), western (Tak, Kanchanaburi, and Prachuap Khiri Khan Provinces), eastern (Chon Buri and Chanthaburi Provinces), northeastern (Nakhon Ratchasima, Khon Kaen, Mukdahan, Nong Khai, Bueng Kan, and Ubon Ratchathani Provinces) and southern (Surat Thani, Phuket, Songkhla, and Narathiwat Provinces) (Table 1 and S1 Table). Soils samples were randomly collected from ten 1-m^2^ sites in each park, with sampling at four positions per site. All soil samples were collected from a depth of approximately 15 cm using a sterile metal spoon to avoid plant debris, weeds, and branches. Samples were placed in sterile plastic bags and stored at 4°C until processed.

**Table 1:** Sample collection areas and number of *Scedosporium apiospermum* species complex strains isolated from each area.

Park name	Park code	Location	Province	No. of sampled sites	No. of isolates	Distribution of *Scedosporium* species complex members		
						*S*. *apiospermum*	*S*. *dehoogii*	Unidentified species	
1. Ko Loi Public Park	CRI-A	N 19.91832; E 099.83858	Chiang Rai	10	0	0	0	0	
2. Anniversary Rama 9 Flag and Lamb Park	CRI-B	N 19.90855; E 099.83505		10	0	0	0	0	
3. Chiang Rai Beach Park	CRI-C	N 19.91714; E 099.79384		10	0	0	0	0	
4. Nong Buak Hard Public Park	CMI-D	N 18.78188; E 098.97968	Chiang Mai	10	0	0	0	0	
5. Lanna Rama 9 Park	CMI-E	N 18.81985; E 098.97861		10	0	0	0	0	
6. Baan Den Health Park	CMI-F	N 18.77238; E 099.00645		10	0	0	0	0	
7. Nong Thin Public Park	NKI-G	N 17.87699; E 102.72742	Nong Khai	10	0	0	0	0	
8. Pumrak Park	NMA-H	N 14.97205; E 102.07129	Nakhon Ratchasima	10	77	76	1	0	
9. Asdang Reservoir Park	NMA-I	N 14.98180; E 102.09743		10	44	44	0	0	
10. Nongkae Chang Park	NMA-J	N 14.96639; E 102.07471		10	33	32	0	1	
11. Paradise Park	NSN-K	N 15.69820; E 100.12359	Nakhon Sawan	10	0	0	0	0	
12. Bueng Phraram Public Park	AYA-L	N 14.35659; E 100.56277	Phra Nakhon Si Ayutthaya	10	34	33	0	1	
13. Chaloem Phrakiat Rama 9 Park (Saun Luang Chonburi)	CBI-M	N 13.33708; E 100.95692	Chonburi	10	0	0	0	0	
14. 80 Anniversary Rama 9 Chaloem Phrakiat Park	CBI-N	N 13.36353; E 100.97855		10	0	0	0	0	
15. Health Park	CBI-O	N 13.36463; E 100.98007		10	0	0	0	0	
16. Somdet Phra Nyanasamvara Park	KRI-P	N 14.02141; E 099.52165	Kanchanaburi	10	0	0	0	0	
17. Municipal Public Park	PKN-Q	N 11.80984; E 099.79892	Prachuap Khiri Khan	10	2	0	0	2	
18. Chaloem Phrakiat Rama 9 Park	PKT-R	N 07.87949; E 098.37398	Phuket	10	0	0	0	0	
19. Free Park (Japan Park)	SKA-S	N 07.21214; E 100.59496		10	0	0	0	0	
20. Courtyard Park	SKA-T	N 07.19175; E 100.59415	Songkhla	10	0	0	0	0	
21. Huai Muang Park	UBN-U	N 15.24694; E 104.84307	Ubon Ratchathani	10	0	0	0	0	
22. Tung Sri Muang Park	UBN-V	N 15.22993; E 104.85844		10	0	0	0	0	
23. Rama 5 Public PArk	NWT-W	N 06.42206; E 101.80879	Narathiwat	10	0	0	0	0	
24. Chaloem Phrakiat Health Park	SKM-X	N 13.411140; E 100.00183	Samut Songkhram	10	1	1	0	0	
25. Municipal Public Park	NYK-Y	N 14.20273; E 101.21530	Nakhon Nayok	10	0	0	0	0	
26. Bueng Kaen Nakhon Public Park	KKN-Z	N 16.41813; E 102.83589	Khon Kaen	10	0	0	0	0	
27. Thanarak Anusorn Public Park	KKN-AA	N 16.43029; E 102.82467		10	0	0	0	0	
28. Bueng Kan Public Park	BKN-AB	N 18.36135; E 103.66105	Bueng Kan	10	0	0	0	0	
29. Somdej Pra Chao Tak Sin Maharat Public Park	CTI-AC	N 12.60178; E 102.10439	Chanthaburi	10	0	0	0	0	
30. Chaloem Phrakiat Rama 9 Public PArk	CTI-AD	N 12.59525; E 102.08495		10	0	0	0	0	
31. Si Mueang Park	NAN-AE	N 18.77608; E 100.77496	Nan	10	0	0	0	0	
32. Chom Nan Chaloem Phrakiat Public Park	PLK-AF	N 16.491.44; E 100.15357	Phitsanulok	10	0	0	0	0	
33. Somdej Phra Naresuan Maharat Shrine Maesod Park	TAK-AG	N 16.43196; E 98.3437	Tak	10	0	0	0	0	
34. Rama 9 Park	SNI-AH	N 9.13820; E 99.34894	Surat Thani	10	0	0	0	0	
35. Mueang Mukdahan Municipal Golden Jubilee Commemoration Public Park	MDH-AI	N 16.54077; E 104.71962	Mukdahan	10	0	0	0	0	
**Total**	**35**	**35**	**23**	**350**	**191**	**188**	**1**	**2**	

### Isolation of *S*. *apiospermum* species complex

Fungal isolation was performed according to the procedure provided by Luplertlop et al. [7]. In brief, 5 g of soil were suspended in 15 ml sterile distilled water, vigorously mixed, and filtered through 100-μm nylon cell strainers (Falcon, Durham, NC, USA). The filtrate was centrifuged at 7,000 ×*g* for 5 min, and the supernatant was discarded. The pellet was then re-suspended in 5 ml distilled water. Three 100-μl aliquots were inoculated on three separate plates of Scedo-Select III medium, which was designed and developed to minimize the growth of other rapidly growing fungal species and specifically for the isolation of *Scedosporium* colonies [8]. The plates were incubated at 35°C for 5 days.

### Morphological identification of *S*. *apiospermum* species complex

Colony morphologies were visually and microscopically examined according to the procedure of Gilgado et al. [9–10]. A single colony of each morphological type on a given plate was selected for further analysis. Colonies identified as *S*. *apiospermum* species complex on the basis of their morphology were inoculated on Scedo-Select III for purification. The colonies isolated from Scedo-Select III plates were then collected and inoculated onto Sabouraud dextrose agar for detailed macroscopic and microscopic observations.

### Molecular identification of *S*. *apiospermum* species complex

Species isolated from soil, as described above, were inoculated in yeast peptone dextrose broth and incubated at 35°C for 7 days. DNA was extracted using the E.Z.N.A. Fungal DNA mini kit (Omega Bio-tek, Norcross, GA, USA) and amplified using PCR with the β-tubulin (Bt2) gene-specific primers Bt2a: 5′GGTAACCAAATCGGTGCTGCTTTC3′ and Bt2b: 5′ACCCTCAGTGTAGTGACCCTTGGC3′ [11]. Each PCR reaction was performed in a 25-μl mixture that contained 0.5 μM of each primer, KAPA 2G Fast HS ReadyMix PCR kit with a loading dye (KAPA Biosystems, USA), nuclease-free water, and genomic DNA using a T100 Thermal Cycler (Bio-Rad) according to the following protocol: 95°C for 6 min, 35 cycles of 95°C for 1 min, 58°C for 1 min, and 72°C for 45 s, and a final extension step of 72°C for 10 min. Then, 5 μl of the PCR product was electrophoresed on a 1.5% agarose gel that contained SERVA DNA Stain G (SERVA Electrophoresis GmbH, Germany) in 1× TBE buffer. Banding patterns were photographed using the Gel Doc XR+ system (Bio-Rad). PCR products (product size ~ 650 bp) were purified using the FavorPrep GEL/PCR Purification Mini Kit (Favorgen Biotech Corporation, Taiwan) and sequenced using gene-specific forward and reverse primers by AITbiotech Pty Ltd (Singapore). The retrieved sequence files were edited and subjected to pairwise alignment using the BioEdit software (http://www.mbio.ncsu.edu/bioedit/bioedit.html). Edited sequences were compared with existing sequences in GenBank using BLASTn (http://blast.ncbi.nlm.nih.gov/Blast.cgi). The generated nucleotide sequences were deposited in GenBank under accession numbers MF991891 and MG204345−MG204534 (The Genbank accession number provided in Supplementary data S1 Table).

### Phylogenetic and genetic diversity analysis

Each sequence was trimmed to the start and end of the gene. The allele number of each sequence was determined using the MLSTest v1.0.1.23 software (downloaded from http://ipe.unsa.edu.ar/software) [12]. Graphic representations of multiple nucleic acid sequence alignments were created using WebLogo 3 (weblogo.threeplusone.com). The best model of evolution was selected from the Bayesian Information Criterion (BIC) in MEGA7 [13]. The model with the lowest BIC score was selected to construct a maximum likelihood phylogenetic tree. A phylogenetic tree of all aligned sequences, excluding gaps, and missing data for the heuristic search was obtained by applying the maximum likelihood approach based on the best model in MEGA7. The tree is drawn to scale, with branch lengths measured in number of substitutions per site. A bootstrap analysis was conducted with 1000 replications, and bootstrap values of ≥50% are shown above the branches. Sequences of reference strains were downloaded from GenBank (S2 Table). All sequences were included in a phylogenetic network of the Bt2 gene created by using the neighbor-net algorithm of SplitsTree4 (downloaded from http://www.splitstree.org/) [14].

### Ethics statement

This field study was performed under permission from the Ministry of Education (letter 0517.115/00331), as well as the following authorities concerned with protection of wildlife: Thanong Donchai, Deputy mayor; Chiangmai Province permitted by Chatri Cheuamanochan, Deputy mayor; Nan Province permitted by Arisa Boonsom, Deputy mayor; Phitsanulok Province permitted by Pongsin Sanepong, Deputy mayor; Phra Nakhon Si Ayutthaya Province permitted by Suvera Rinvet, Municipal Clerk; Samut Songkhram Province permitted by Somchai Tonpasert, Mayor; Tak Province permitted by Ananchai Taweeguekolkit, Mayor; Kanchanaburi Province permitted by Kumtorn Pusitkarnchana, Deputy mayor; Prachuap Khiri Khan Province permitted by Pichit Santimethakul, Deputy mayor; Chon Buri permitted by Jutharat Parinwachirapat, Municipal Clerk; Chanthaburi Province permitted by Pirat Atikarnkul, Municipal Clerk; Nakhon Ratchasima Province permitted by Nutcharat Chuhirunwat, Director of Public Works; Khon Kean Province permitted by Thawatchai Wanapithakkul, Mayor; Mukdahan Province permitted by Trirong Thawinprai, Deputy mayor; Nong Khai Province permitted by Winchai Waipat, Deputy mayor; Bueng Kan Province permitted by Witaya Saynjanthichai, Mayor; Ubon Ratchathani Province permitted by Kritchapol Muangnue, Deputy mayor; Surat Thani Province permitted by Suphang Saewong, Deputy mayor; Phuket Province permitted by Thavorn Jirapatsophon, Deputy mayor; Songkhla Province pemitted by Somsak Tanthiseranee, Mayor and Narathiwat Province permitted by Udom Densanthikul, Mayor. The field study did not involve endangered or protected species.

## Results

**Fig 1:**
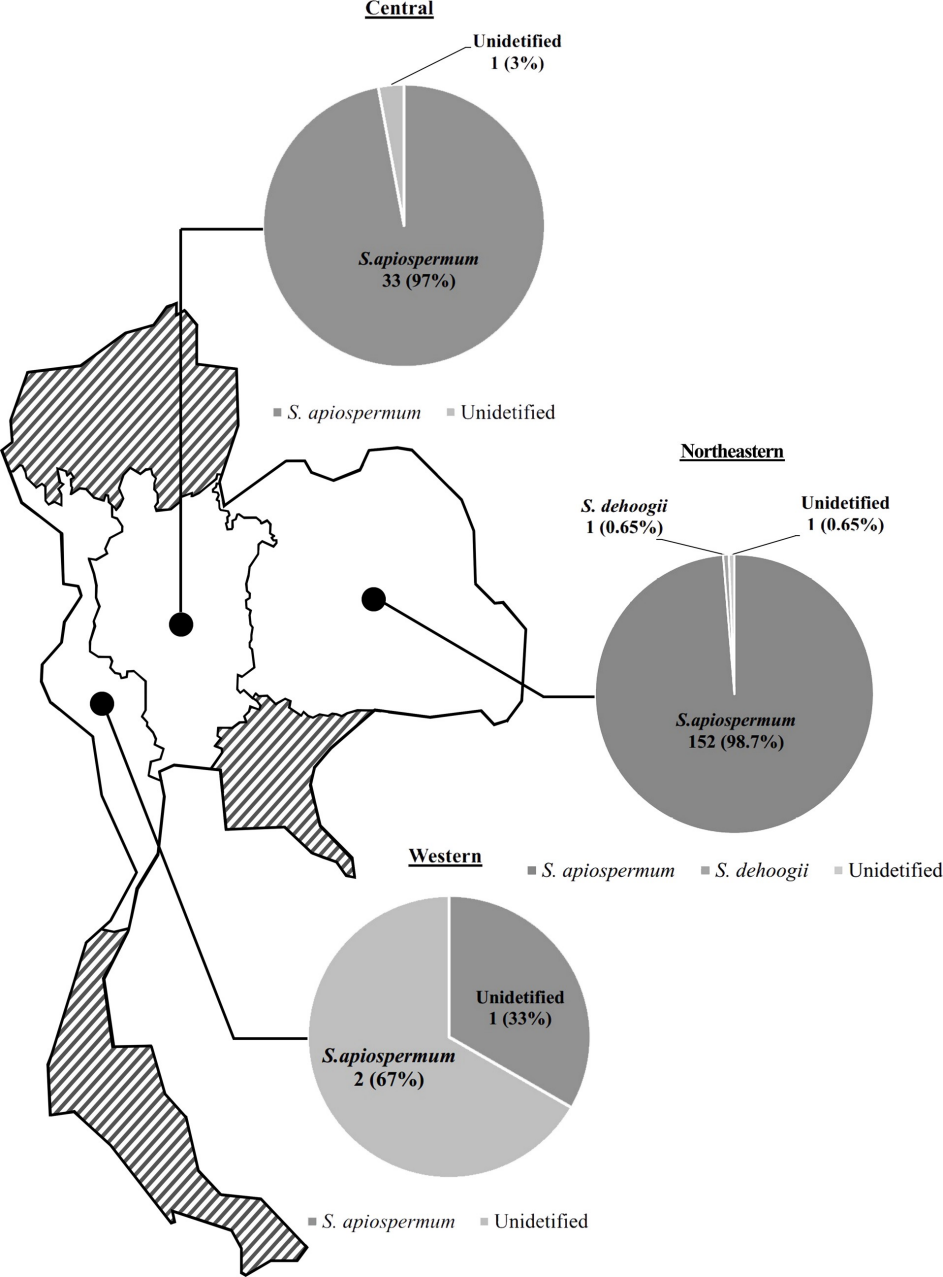
The distribution of *Scedosporium* species across six regions of Thailand.

In this study, 350 soil samples were collected from 35 public parks that were distributed across 23 provinces of Thailand (Table 1). In total, 191 morphologically distinct colonies of *Scedosporium* were selected for species and strain identification based on Bt2 gene sequencing. Of the 191 isolates, 188 were *S*. *apiospermum* (76 isolates from Pumrak Park, 44 isolates from Asdang Park, and 32 isolates from Nongkae Chang Park in Nakhon Ratchasima Province; 33 isolates from Bueng Phraram Public Park in Phra Nakhon Si Ayutthaya Province; one isolate from Chaloem Phrakiat Health Park in Samut Songkhram Province; and two isolates from Municipal Public Park in Prachuap Khiri Khan Province), and one isolate of *S*. *dehoogii* (TMMI154; isolated from Pumrak Park in Nakhon Ratchasima Province) as summarized in Fig 1. In addition, the species of two isolates (TMMI275 from Nongkae Chang Park in Nakhon Ratchasima Province and TMMI293 from Bueng Phraram Public Park in Phra Nakhon Si Ayutthaya Province) could not be identified.

**Fig 2:**
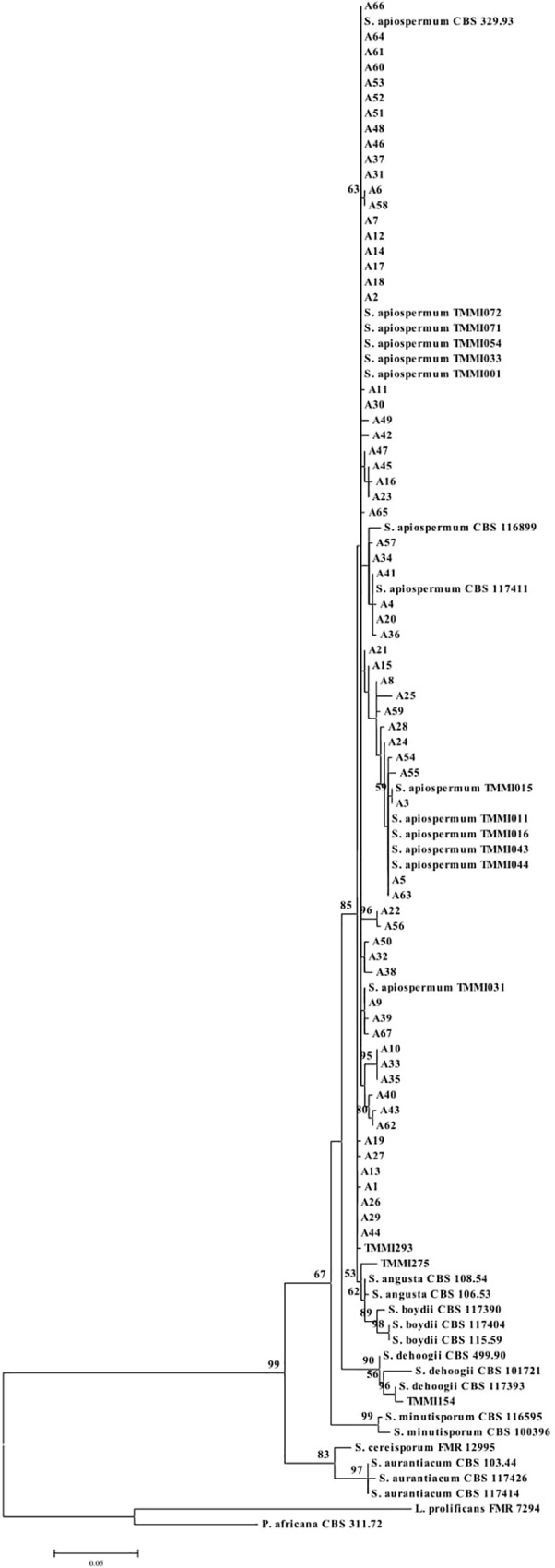
Molecular phylogenetic maximum likelihood analysis of the Bt2 gene. A1−A67 are the allele types.

Given the large number of *S*. *apiospermum* sequences, we determined the number of Bt2 allele types according to sequence similarity before multiple alignment. From the 188 sequences, we found 67 alleles, with allele frequencies ranging from 1 to 34 individuals sampled (Table 2). A graphic representation of the multiple nucleic acid sequence alignment for these 67 allele types was created, and a phylogenetic tree was constructed (Fig 2). The best model for Bt2 gene analyses was the K2+G model (K2: Kimura-2-Parameter; +G: Gamma distribution), with the BIC score of 6327.810. Therefore, the maximum likelihood phylogenetic tree of the concatenated data set was created based on the K2+G model. A discrete gamma distribution was used to model the evolutionary rate differences among sampling sites [five categories (+G, parameter = 0.0500)].

**Table 2:** Alleles (A), their frequencies, and the strains with each allele.

A	Frequency	Strains (Genbank accession number)
1	15	TMMI111 (MG204346), TMMI112 (MG204347), TMMI113 (MG204348), TMMI114 (MG204349), TMMI115 (MG204349), TMMI116 (MG204351), TMMI118 (MG204353), TMMI119 (MG204354), TMMI123 (MG204358), TMMI124 (MG204359), TMMI126 (MG204361), TMMI128 (MG204363), TMMI129 (MG204364), TMMI130 (MG204365), TMMI155 (MG204389)
2	34	TMMI117 (MG204352), TMMI120 (MG204355), TMMI122 (MG204357), TMMI137 (MG204372), TMMI138 (MG204373), TMMI143 (MG204378), TMMI150 (MG204385), TMMI158 (MG204392), TMMI160 (MG204394), TMMI166 (MG204400), TMMI172 (MG204406), TMMI175 (MG204409), TMMI182 (MG204416), TMMI190 (MG204424), TMMI191 (MG204425), TMMI201 (MG204435), TMMI203 (MG204437), TMMI204 (MG204438), TMMI208 (MG204442), TMMI210 (MG204444), TMMI211 (MG204445), TMMI219 (MG204453), TMMI221 (MG204455), TMMI223 (MG204457), TMMI246 (MG204480), TMMI248 (MG204482), TMMI250 (MG204484), TMMI253 (MG204487), TMMI256 (MG204490), TMMI272 (MG204506), TMMI274 (MG204508), TMMI283 (MG204516), TMMI294 (MG204527), TMMI296 (MG204529)
3	2	TMMI121 (MG204356), TMMI225 (MG204459)
4	1	TMMI125 (MG204360)
5	17	TMMI127 (MG204362), TMMI132 (MG204367), TMMI141 (MG204376), TMMI163 (MG204397), TMMI168 (MG204402), TMMI185 (MG204419), TMMI243 (MG204477), TMMI247 (MG204481), TMMI251 (MG204485), TMMI252 (MG204486), TMMI261 (MG204495), TMMI262 (MG204496), TMMI263 (MG204497), TMMI280 (MG204513), TMMI281 (MG204514), TMMI285 (MG204518), TMMI292 (MG204525)
6	2	TMMI131 (MG204366), TMMI268 (MG204502)
7	14	TMMI133 (MG204368), TMMI153 (MG204388), TMMI161 (MG204395), TMMI179 (MG204413), TMMI238 (MG204472), TMMI239 (MG204473), TMMI244 (MG204478), TMMI249 (MG204483), TMMI257 (MG204491), TMMI270 (MG204504), TMMI271 (MG204505), TMMI282 (MG204515), TMMI286 (MG204519), TMMI301 (MG204534)
8	3	TMMI134 (MG204369), TMMI139 (MG204374), TMMI145 (MG204380)
9	15	TMMI135 (MG204370), TMMI140 (MG204375), TMMI142 (MG204377), TMMI144 (MG204379), TMMI146 (MG204381), TMMI148 (MG204383), TMMI149 (MG204384), TMMI152 (MG204387), TMMI162 (MG204396), TMMI216 (MG204450), TMMI218 (MG204452), TMMI224 (MG204458), TMMI226 (MG204460), TMMI258 (MG204492), TMMI287 (MG204520)
10	2	TMMI136 (MG204371), TMMI197 (MG204431)
11	1	TMMI147 (MG204382)
12	1	TMMI151 (MG204386)
13	6	TMMI156 (MG204390), TMMI157 (MG204391), TMMI213 (MG204447), TMMI214 (MG204448), TMMI284 (MG204517), TMMI289 (MG204522)
14	1	TMMI159 (MG204393)
15	4	TMMI164 (MG204398), TMMI167 (MG204401), TMMI236 (MG204470), TMMI242 (MG204476)
16	1	TMMI165 (MG204399)
17	1	TMMI169 (MG204403)
18	1	TMMI170 (MG204404)
19	1	TMMI171 (MG204405)
20	6	TMMI173 (MG204407), TMMI188 (MG204422), TMMI194 (MG204428), TMMI220 (MG204454), TMMI265 (MG204499), TMMI290 (MG204523)
21	1	TMMI174 (MG204408)
22	1	TMMI176 (MG204410)
23	2	TMMI177 (MG204411), TMMI259 (MG204493)
24	2	TMMI178 (MG204412), TMMI267 (MG204501)
25	1	TMMI180 (MG204414)
26	1	TMMI181 (MG201145)
27	1	TMMI183 (MG204417)
28	1	TMMI184 (MG204418)
29	1	TMMI186 (MG204420)
30	1	TMMI187 (MG204421)
31	2	TMMI189 (MG204423), TMMI195 (MG204429)
32	6	TMMI192 (MG204426), TMMI193 (MG204427), TMMI198 (MG204432), TMMI199 (MG204433), TMMI278 (MG204511), TMMI298 (MG204531)
33	1	TMMI196 (MG204430)
34	1	TMMI200 (MG204434)
35	1	TMMI202 (MG204436)
36	1	TMMI205 (MG204439)
37	1	TMMI206 (MG204440)
38	1	TMMI207 (MG204441)
39	1	TMMI209 (MG204443)
40	1	TMMI212 (MG204446)
41	2	TMMI215 (MG204449), TMMI254 (MG204488)
42	1	TMMI217 (MG204451)
43	1	TMMI222 (MG204456)
44	1	TMMI227 (MG204461)
45	2	TMMI228 (MG204462), TMMI240 (MG204474)
46	1	TMMI229 (MG204463)
47	1	TMMI230 (MG204464)
48	1	TMMI231 (MG204465)
49	1	TMMI232 (MG204466)
50	1	TMMI233 (MG204467)
51	1	TMMI234 (MG204468)
52	1	TMMI235 (MG204469)
53	2	TMMI237 (MG204471), TMMI245 (MG204479)
54	1	TMMI241 (MG204475)
55	1	TMMI255 (MG204489)
56	1	TMMI260 (MG204494)
57	1	TMMI264 (MG204498)
58	3	TMMI266 (MG204500), TMMI291 (MG204524), TMMI297 (MG204530)
59	1	TMMI269 (MG204503)
60	1	TMMI273 (MG204507)
61	1	TMMI276 (MG204509)
62	1	TMMI277 (MG204510)
63	1	TMMI279 (MG204512)
64	1	TMMI288 (MG204521)
65	1	TMMI295 (MG204528)
66	1	TMMI299 (MG204532)
67	1	TMMI300 (MG204533)

**Fig 3:**
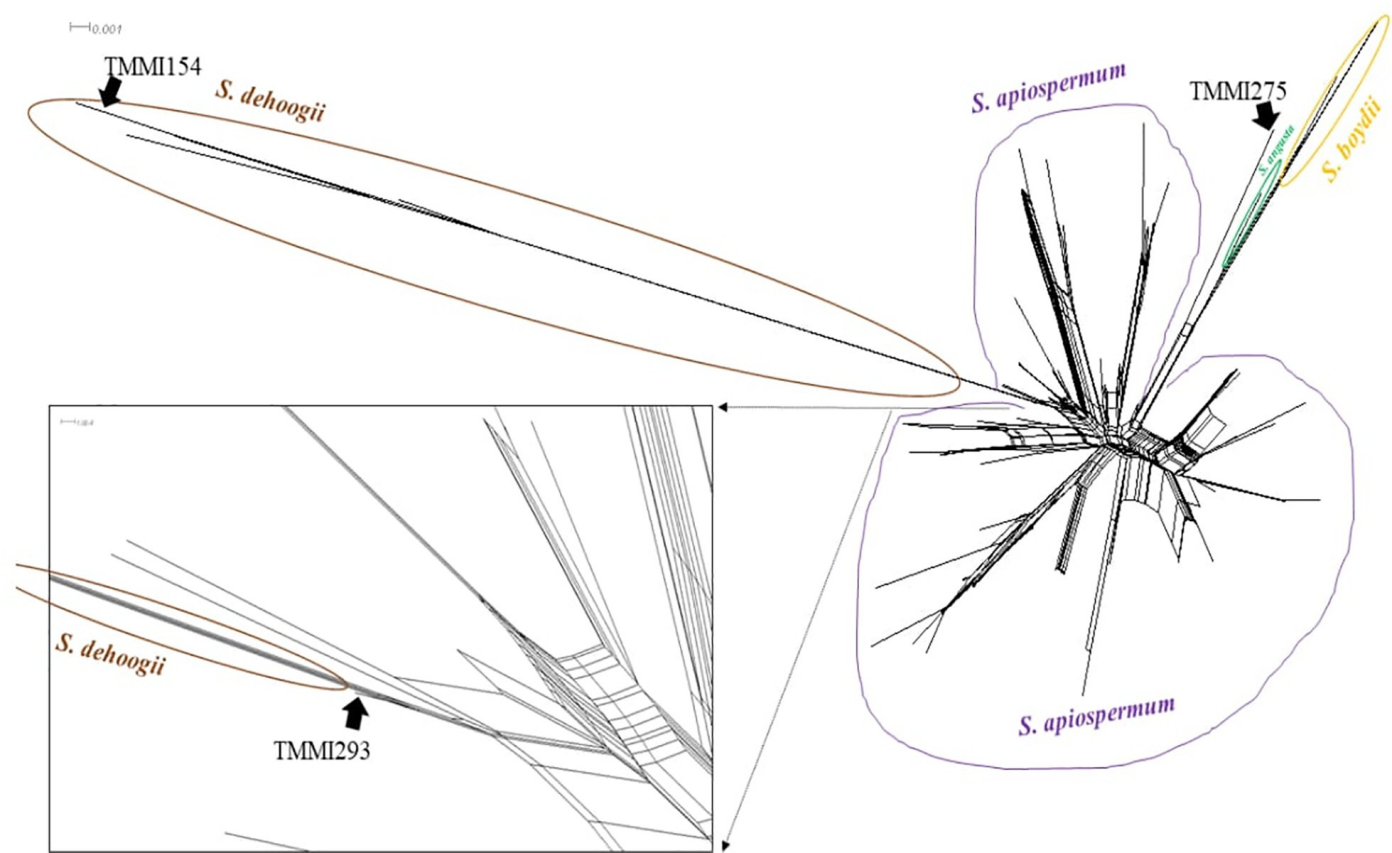
Phylogenetic network. SplitTree decomposition analysis using the neighbor-net algorithm for the Bt2 gene.

We then performed a genetic diversity analysis using the Splits Tree algorithm. Fig 3 shows the genetic associations among all 191 genotyped variants of the Bt2 gene. Strain TMMI154 was clustered with *S*. *dehoogii*, whereas TMMI275 and TMMI293 were alone in the Bt2 gene parallelogram, suggesting new species.

## Discussion

There are several molecular techniques applied to identify *Scedosporium* species. Sequencing techniques, including Multi-Locus Sequence Typing (MLST), is widely used for identification of these fungi. The target gene using for sequencing are internal transcribed spacer (ITS), actin, calmodulin exons 3 and 4, the second largest subunit of the RNA polymerase II and Beta-tubulin exon 2–4, manganese superoxide dismutase, transcription elongation 1 alpha, Beta -tubulin exon 5–6 [15]. Researchers have also used the internal transcribe spacer-restriction fragment length polymorphism (ITS-RFLP) technique, M13 PCR fingerprint, quantitative Real-Time PCR (qPCR), PCR-based reverse line blotting (PCR-RLB) and loop-mediated isothermal amplification (LAMP) [16–17]. Isolates should be characterized phenotypically as well as with molecular techniques to confirm *Scedosporium* species identification.

Based on molecular variation, the *S*. *aspiospermum* species complex is comprised of *S*. *apiospermum*, *S*. *boydii*, *S*. *dehoogii*, *S*. *aurantiacum*, and *S*. *minutisporum* (*Pseudallescheria minutispora*) according to the European Confederation of Medical Mycology/International Society for Human and Animal Mycology [1]. More recently, a new species, *S*. *cereisporum*, was isolated by Rougeron et al.; the species was phylogenetically and morphologically closely related to *S*. *aurantiacum* [18]. Chen et al. [19] defined the *S*. *apiospermum* species complex as *S*. *apiospermum*, *S*. *boydii*, and *S*. *angusta* (*P*. *angusta*) based on the phylogenetic analysis of BT2, γ-actin, transcriptional elongation factor 1α, and internal transcribed spacer of the small ribosomal protein 60sS L10 (L1), which distinguished *S*. *minutisporum*, *S*. *aurantiacum*, and *S*. *dehoogii* from the three *S*. *apiospermum* species complex. There are currently >10 species of the *S*. *apiospermum* species complex [1]. Furthermore, at least five are pathogenic, with infection cases reported in Thailand and across Asia. Therefore, we examined *Scedosporium* species distribution in the present study.

In addition to *S*. *apiospermum*, *S*. *boydii* infection has been documented in Thailand. *S*. *boydii* was specifically isolated from the brain tissue of a renal transplant patient. In the present study, samples from only four of 23 provinces (Nakhon Ratchasima, Phra Nakhon Si Ayutthaya, Samut Songkhram, and Prajuap Khiri Khan) were positive for *Scedosporium* species; however, at these sites, numerous *S*. *apiospermum* strains (188 from 191 isolates) were found. In addition, one isolate of *S*. *dehoogii* was identified, whereas two isolates were unidentifiable and thus could be novel species. The two unclassified strains, TMMI 275 and TMMI 293, were closely related to *S*. *angusta* and *S*. *apiospermum*, respectively. The present study confirms that *S*. *apiospermum* inhabits soil in diverse regions of Thailand (northeastern, central, and western), and *S*. *aurantiacum* was only isolated from a single site around Bangkok.

Therefore, this study demonstrated that *Scedosporium* distribution for guiding the further perspective in factor related fungal distribution and an impact. Our survey data indicate the prevalence of *Scedosporium* in different regions of Thailand, which should be useful for the clinician and medical mycologist in diagnosing fungal infections. This information may help clinicians to exclude *Aspergillus* as a cause of fungal infection.

## Conclusion

In conclusion, this study identified soil as an ecological niche of *Scedosporium* in Thailand. The results provide valuable knowledge to assist future studies to compare genetic relatedness among pathogenic species in the clinical setting and to evaluate infection risk in specific regions.

## Supporting information

S1 Table The information and place of fungal isolated. (PDF)

S2 Table Sequences of the reference strains (download from GenBank). (PDF)
