# Supplementary material for: Distribution of Scedosporium species in soil from areas with high human population density and tourist popularity in six geographic regions in Thailand
Source: PLoS One. 2019 Jan 23;14(1):e0210942. doi: 10.1371/journal.pone.0210942 (PMC6343921; doi:10.1371/journal.pone.0210942)
Supplement: S1 Table — (PDF) [file pone.0210942.s001.pdf]

S1 Table. The information and place of fungal isolated.

	ID	Isolate name	Sequencing	Genbank Accesion	Park Name	Province
1	TMMI111	H11	*Scedosporium apiospermum*	MG204346	Pumrak Park	Nakhon Rachasima/North-Eastern
2	TMMI112	H12	*Scedosporium apiospermum*	MG204347	Pumrak Park	Nakhon Rachasima/North-Eastern
3	TMMI113	H14	*Scedosporium apiospermum*	MG204348	Pumrak Park	Nakhon Rachasima/North-Eastern
4	TMMI114	H15	*Scedosporium apiospermum*	MG204349	Pumrak Park	Nakhon Rachasima/North-Eastern
5	TMMI115	H16	*Scedosporium apiospermum*	MG204350	Pumrak Park	Nakhon Rachasima/North-Eastern
6	TMMI116	H17	*Scedosporium apiospermum*	MG204351	Pumrak Park	Nakhon Rachasima/North-Eastern
7	TMMI117	H18	*Scedosporium apiospermum*	MG204352	Pumrak Park	Nakhon Rachasima/North-Eastern
8	TMMI118	H19	*Scedosporium apiospermum*	MG204353	Pumrak Park	Nakhon Rachasima/North-Eastern
9	TMMI119	H110	*Scedosporium apiospermum*	MG204354	Pumrak Park	Nakhon Rachasima/North-Eastern
10	TMMI120	H111	*Scedosporium apiospermum*	MG204355	Pumrak Park	Nakhon Rachasima/North-Eastern
11	TMMI121	H112	*Scedosporium apiospermum*	MG204356	Pumrak Park	Nakhon Rachasima/North-Eastern
12	TMMI122	H113	*Scedosporium apiospermum*	MG204357	Pumrak Park	Nakhon Rachasima/North-Eastern
13	TMMI123	H114	*Scedosporium apiospermum*	MG204358	Pumrak Park	Nakhon Rachasima/North-Eastern
14	TMMI124	H115	*Scedosporium apiospermum*	MG204359	Pumrak Park	Nakhon Rachasima/North-Eastern
15	TMMI125	H116	*Scedosporium apiospermum*	MG204360	Pumrak Park	Nakhon Rachasima/North-Eastern
16	TMMI126	H117	*Scedosporium apiospermum*	MG204361	Pumrak Park	Nakhon Rachasima/North-Eastern
17	TMMI127	H118	*Scedosporium apiospermum*	MG204362	Pumrak Park	Nakhon Rachasima/North-Eastern
18	TMMI128	H119	*Scedosporium apiospermum*	MG204363	Pumrak Park	Nakhon Rachasima/North-Eastern
19	TMMI129	H120	*Scedosporium apiospermum*	MG204364	Pumrak Park	Nakhon Rachasima/North-Eastern
20	TMMI130	H121	*Scedosporium apiospermum*	MG204365	Pumrak Park	Nakhon Rachasima/North-Eastern
21	TMMI131	H31	*Scedosporium apiospermum*	MG204366	Pumrak Park	Nakhon Rachasima/North-Eastern
22	TMMI132	H32	*Scedosporium apiospermum*	MG204367	Pumrak Park	Nakhon Rachasima/North-Eastern
23	TMMI133	H33	*Scedosporium apiospermum*	MG204368	Pumrak Park	Nakhon Rachasima/North-Eastern
24	TMMI134	H41	*Scedosporium apiospermum*	MG204369	Pumrak Park	Nakhon Rachasima/North-Eastern
25	TMMI135	H42	*Scedosporium apiospermum*	MG204370	Pumrak Park	Nakhon Rachasima/North-Eastern
26	TMMI136	H43	*Scedosporium apiospermum*	MG204371	Pumrak Park	Nakhon Rachasima/North-Eastern
27	TMMI137	H44	*Scedosporium apiospermum*	MG204372	Pumrak Park	Nakhon Rachasima/North-Eastern

28	TMMI138	H45	*Scedosporium apiospermum*	MG204373	Pumrak Park	Nakhon Rachasima/North-Eastern
29	TMMI139	H46	*Scedosporium apiospermum*	MG204374	Pumrak Park	Nakhon Rachasima/North-Eastern
30	TMMI140	H47	*Scedosporium apiospermum*	MG204375	Pumrak Park	Nakhon Rachasima/North-Eastern
31	TMMI141	H48	*Scedosporium apiospermum*	MG204376	Pumrak Park	Nakhon Rachasima/North-Eastern
32	TMMI142	H49	*Scedosporium apiospermum*	MG204377	Pumrak Park	Nakhon Rachasima/North-Eastern
33	TMMI143	H410	*Scedosporium apiospermum*	MG204378	Pumrak Park	Nakhon Rachasima/North-Eastern
34	TMMI144	H411	*Scedosporium apiospermum*	MG204379	Pumrak Park	Nakhon Rachasima/North-Eastern
35	TMMI145	H412	*Scedosporium apiospermum*	MG204380	Pumrak Park	Nakhon Rachasima/North-Eastern
36	TMMI146	H413	*Scedosporium apiospermum*	MG204381	Pumrak Park	Nakhon Rachasima/North-Eastern
37	TMMI147	H414	*Scedosporium apiospermum*	MG204382	Pumrak Park	Nakhon Rachasima/North-Eastern
38	TMMI148	H415	*Scedosporium apiospermum*	MG204383	Pumrak Park	Nakhon Rachasima/North-Eastern
39	TMMI149	H416	*Scedosporium apiospermum*	MG204384	Pumrak Park	Nakhon Rachasima/North-Eastern
40	TMMI150	H417	*Scedosporium apiospermum*	MG204385	Pumrak Park	Nakhon Rachasima/North-Eastern
41	TMMI151	H418	*Scedosporium apiospermum*	MG204386	Pumrak Park	Nakhon Rachasima/North-Eastern
42	TMMI152	H51	*Scedosporium apiospermum*	MG204387	Pumrak Park	Nakhon Rachasima/North-Eastern
43	TMMI153	H52	*Scedosporium apiospermum*	MG204388	Pumrak Park	Nakhon Rachasima/North-Eastern
44	TMMI154	H53	*Scedosporium dehoogii*	MF991891	Pumrak Park	Nakhon Rachasima/North-Eastern
45	TMMI155	H54	*Scedosporium apiospermum*	MG204389	Pumrak Park	Nakhon Rachasima/North-Eastern
46	TMMI156	H55	*Scedosporium apiospermum*	MG204390	Pumrak Park	Nakhon Rachasima/North-Eastern
47	TMMI157	H56	*Scedosporium apiospermum*	MG204391	Pumrak Park	Nakhon Rachasima/North-Eastern
48	TMMI158	H94	*Scedosporium apiospermum*	MG204392	Pumrak Park	Nakhon Rachasima/North-Eastern
49	TMMI159	H104	*Scedosporium apiospermum*	MG204393	Pumrak Park	Nakhon Rachasima/North-Eastern
50	TMMI160	H109	*Scedosporium apiospermum*	MG204394	Pumrak Park	Nakhon Rachasima/North-Eastern
51	TMMI161	H1010	*Scedosporium apiospermum*	MG204395	Pumrak Park	Nakhon Rachasima/North-Eastern
52	TMMI162	H1018	*Scedosporium apiospermum*	MG204396	Pumrak Park	Nakhon Rachasima/North-Eastern
53	TMMI163	I110	*Scedosporium apiospermum*	MG204397	Asdang Park	Nakhon Rachasima/North-Eastern
54	TMMI164	I111	*Scedosporium apiospermum*	MG204398	Asdang Park	Nakhon Rachasima/North-Eastern
55	TMMI165	I112	*Scedosporium apiospermum*	MG204399	Asdang Park	Nakhon Rachasima/North-Eastern
56	TMMI166	I113	*Scedosporium apiospermum*	MG204400	Asdang Park	Nakhon Rachasima/North-Eastern
57	TMMI167	I114	*Scedosporium apiospermum*	MG204401	Asdang Park	Nakhon Rachasima/North-Eastern
58	TMMI168	I115	*Scedosporium apiospermum*	MG204402	Asdang Park	Nakhon Rachasima/North-Eastern

59	TMMI169	I117	*Scedosporium apiospermum*	MG204403	Asdang Park	Nakhon Rachasima/North-Eastern
60	TMMI170	I118	*Scedosporium apiospermum*	MG204404	Asdang Park	Nakhon Rachasima/North-Eastern
61	TMMI171	I119	*Scedosporium apiospermum*	MG204405	Asdang Park	Nakhon Rachasima/North-Eastern
62	TMMI172	I615	*Scedosporium apiospermum*	MG204406	Asdang Park	Nakhon Rachasima/North-Eastern
63	TMMI173	I617	*Scedosporium apiospermum*	MG204407	Asdang Park	Nakhon Rachasima/North-Eastern
64	TMMI174	I619	*Scedosporium apiospermum*	MG204408	Asdang Park	Nakhon Rachasima/North-Eastern
65	TMMI175	I623	*Scedosporium apiospermum*	MG204409	Asdang Park	Nakhon Rachasima/North-Eastern
66	TMMI176	I624	*Scedosporium apiospermum*	MG204410	Asdang Park	Nakhon Rachasima/North-Eastern
67	TMMI177	I626	*Scedosporium apiospermum*	MG204411	Asdang Park	Nakhon Rachasima/North-Eastern
68	TMMI178	I628	*Scedosporium apiospermum*	MG204412	Asdang Park	Nakhon Rachasima/North-Eastern
69	TMMI179	J29	*Scedosporium apiospermum*	MG204413	Nongkae Chang Park	Nakhon Rachasima/North-Eastern
70	TMMI180	J43	*Scedosporium apiospermum*	MG204414	Nongkae Chang Park	Nakhon Rachasima/North-Eastern
71	TMMI181	J45	*Scedosporium apiospermum*	MG204415	Nongkae Chang Park	Nakhon Rachasima/North-Eastern
72	TMMI182	J46	*Scedosporium apiospermum*	MG204416	Nongkae Chang Park	Nakhon Rachasima/North-Eastern
73	TMMI183	J48	*Scedosporium apiospermum*	MG204417	Nongkae Chang Park	Nakhon Rachasima/North-Eastern
74	TMMI184	J73	*Scedosporium apiospermum*	MG204418	Nongkae Chang Park	Nakhon Rachasima/North-Eastern
75	TMMI185	J74	*Scedosporium apiospermum*	MG204419	Nongkae Chang Park	Nakhon Rachasima/North-Eastern
76	TMMI186	J76	*Scedosporium apiospermum*	MG204420	Nongkae Chang Park	Nakhon Rachasima/North-Eastern
77	TMMI187	J85	*Scedosporium apiospermum*	MG204421	Nongkae Chang Park	Nakhon Rachasima/North-Eastern
78	TMMI188	J87	*Scedosporium apiospermum*	MG204422	Nongkae Chang Park	Nakhon Rachasima/North-Eastern
79	TMMI189	J101	*Scedosporium apiospermum*	MG204423	Nongkae Chang Park	Nakhon Rachasima/North-Eastern
80	TMMI190	J102	*Scedosporium apiospermum*	MG204424	Nongkae Chang Park	Nakhon Rachasima/North-Eastern
81	TMMI191	J103	*Scedosporium apiospermum*	MG204425	Nongkae Chang Park	Nakhon Rachasima/North-Eastern
82	TMMI192	J106	*Scedosporium apiospermum*	MG204426	Nongkae Chang Park	Nakhon Rachasima/North-Eastern
83	TMMI193	J107	*Scedosporium apiospermum*	MG204427	Nongkae Chang Park	Nakhon Rachasima/North-Eastern
84	TMMI194	J211	*Scedosporium apiospermum*	MG204428	Nongkae Chang Park	Nakhon Rachasima/North-Eastern
85	TMMI195	J1010	*Scedosporium apiospermum*	MG204429	Nongkae Chang Park	Nakhon Rachasima/North-Eastern
86	TMMI196	L28	*Scedosporium apiospermum*	MG204430	Bueng Phraram Public Park	Pra Nakhobn Sri Ayuthaya/Central
87	TMMI197	L29	*Scedosporium apiospermum*	MG204431	Bueng Phraram Public Park	Pra Nakhobn Sri Ayuthaya/Central

88	TMMI198	L210	*Scedosporium apiospermum*	MG204432	Bueng Phraram Public Park	Pra Nakhobn Sri Ayuthaya/Central
89	TMMI199	L212	*Scedosporium apiospermum*	MG204433	Bueng Phraram Public Park	Pra Nakhobn Sri Ayuthaya/Central
90	TMMI200	L213	*Scedosporium apiospermum*	MG204434	Bueng Phraram Public Park	Pra Nakhobn Sri Ayuthaya/Central
91	TMMI201	L214	*Scedosporium apiospermum*	MG204435	Bueng Phraram Public Park	Pra Nakhobn Sri Ayuthaya/Central
92	TMMI202	H108	*Scedosporium apiospermum*	MG204436	Pumrak Park	Nakhon Rachasima/North-Eastern
93	TMMI203	H91	*Scedosporium apiospermum*	MG204437	Pumrak Park	Nakhon Rachasima/North-Eastern
94	TMMI204	H93	*Scedosporium apiospermum*	MG204438	Pumrak Park	Nakhon Rachasima/North-Eastern
95	TMMI205	H101	*Scedosporium apiospermum*	MG204439	Pumrak Park	Nakhon Rachasima/North-Eastern
96	TMMI206	H102	*Scedosporium apiospermum*	MG204440	Pumrak Park	Nakhon Rachasima/North-Eastern
97	TMMI207	H103	*Scedosporium apiospermum*	MG204441	Pumrak Park	Nakhon Rachasima/North-Eastern
98	TMMI208	H105	*Scedosporium apiospermum*	MG204442	Pumrak Park	Nakhon Rachasima/North-Eastern
99	TMMI209	H106	*Scedosporium apiospermum*	MG204443	Pumrak Park	Nakhon Rachasima/North-Eastern
100	TMMI210	H107	*Scedosporium apiospermum*	MG204444	Pumrak Park	Nakhon Rachasima/North-Eastern
101	TMMI211	H1011	*Scedosporium apiospermum*	MG204445	Pumrak Park	Nakhon Rachasima/North-Eastern
102	TMMI212	H1012	*Scedosporium apiospermum*	MG204446	Pumrak Park	Nakhon Rachasima/North-Eastern
103	TMMI213	H1013	*Scedosporium apiospermum*	MG204447	Pumrak Park	Nakhon Rachasima/North-Eastern
104	TMMI214	H1014	*Scedosporium apiospermum*	MG204448	Pumrak Park	Nakhon Rachasima/North-Eastern
105	TMMI215	H1015	*Scedosporium apiospermum*	MG204449	Pumrak Park	Nakhon Rachasima/North-Eastern
106	TMMI216	H1016	*Scedosporium apiospermum*	MG204450	Pumrak Park	Nakhon Rachasima/North-Eastern
107	TMMI217	H1017	*Scedosporium apiospermum*	MG204451	Pumrak Park	Nakhon Rachasima/North-Eastern
108	TMMI218	H1019	*Scedosporium apiospermum*	MG204452	Pumrak Park	Nakhon Rachasima/North-Eastern
109	TMMI219	H1020	*Scedosporium apiospermum*	MG204453	Pumrak Park	Nakhon Rachasima/North-Eastern
110	TMMI220	H1021	*Scedosporium apiospermum*	MG204454	Pumrak Park	Nakhon Rachasima/North-Eastern
111	TMMI221	H1022	*Scedosporium apiospermum*	MG204455	Pumrak Park	Nakhon Rachasima/North-Eastern
112	TMMI222	H1023	*Scedosporium apiospermum*	MG204456	Pumrak Park	Nakhon Rachasima/North-Eastern
113	TMMI223	H1024	*Scedosporium apiospermum*	MG204457	Pumrak Park	Nakhon Rachasima/North-Eastern
114	TMMI224	H1025	*Scedosporium apiospermum*	MG204458	Pumrak Park	Nakhon Rachasima/North-Eastern

115	TMMI225	H1026	*Scedosporium apiospermum*	MG204459	Pumrak Park	Nakhon Rachasima/North-Eastern
116	TMMI226	I14	*Scedosporium apiospermum*	MG204460	Asdang Park	Nakhon Rachasima/North-Eastern
117	TMMI227	I15	*Scedosporium apiospermum*	MG204461	Asdang Park	Nakhon Rachasima/North-Eastern
118	TMMI228	I16	*Scedosporium apiospermum*	MG204462	Asdang Park	Nakhon Rachasima/North-Eastern
119	TMMI229	H92	*Scedosporium apiospermum*	MG204463	Pumrak Park	Nakhon Rachasima/North-Eastern
120	TMMI230	I12	*Scedosporium apiospermum*	MG204464	Asdang Park	Nakhon Rachasima/North-Eastern
121	TMMI231	L22	*Scedosporium apiospermum*	MG204465	Bueng Phraram Public Park	Pra Nakhobn Sri Ayuthaya/Central
122	TMMI232	L23	*Scedosporium apiospermum*	MG204466	Bueng Phraram Public Park	Pra Nakhobn Sri Ayuthaya/Central
123	TMMI233	L24	*Scedosporium apiospermum*	MG204467	Bueng Phraram Public Park	Pra Nakhobn Sri Ayuthaya/Central
124	TMMI234	L25	*Scedosporium apiospermum*	MG204468	Bueng Phraram Public Park	Pra Nakhobn Sri Ayuthaya/Central
125	TMMI235	L26	*Scedosporium apiospermum*	MG204469	Bueng Phraram Public Park	Pra Nakhobn Sri Ayuthaya/Central
126	TMMI236	I13	*Scedosporium apiospermum*	MG204470	Asdang Park	Nakhon Rachasima/North-Eastern
127	TMMI237	I17	*Scedosporium apiospermum*	MG204471	Asdang Park	Nakhon Rachasima/North-Eastern
128	TMMI238	I18	*Scedosporium apiospermum*	MG204472	Asdang Park	Nakhon Rachasima/North-Eastern
129	TMMI239	I19	*Scedosporium apiospermum*	MG204473	Asdang Park	Nakhon Rachasima/North-Eastern
130	TMMI240	I116	*Scedosporium apiospermum*	MG204474	Asdang Park	Nakhon Rachasima/North-Eastern
131	TMMI241	I120	*Scedosporium apiospermum*	MG204475	Asdang Park	Nakhon Rachasima/North-Eastern
132	TMMI242	I41	*Scedosporium apiospermum*	MG204476	Asdang Park	Nakhon Rachasima/North-Eastern
133	TMMI243	I51	*Scedosporium apiospermum*	MG204477	Asdang Park	Nakhon Rachasima/North-Eastern
134	TMMI244	I52	*Scedosporium apiospermum*	MG204478	Asdang Park	Nakhon Rachasima/North-Eastern
135	TMMI245	I62	*Scedosporium apiospermum*	MG204479	Asdang Park	Nakhon Rachasima/North-Eastern
136	TMMI246	I65	*Scedosporium apiospermum*	MG204480	Asdang Park	Nakhon Rachasima/North-Eastern
137	TMMI247	I66	*Scedosporium apiospermum*	MG204481	Asdang Park	Nakhon Rachasima/North-Eastern
138	TMMI248	I67	*Scedosporium apiospermum*	MG204482	Asdang Park	Nakhon Rachasima/North-Eastern
139	TMMI249	I68	*Scedosporium apiospermum*	MG204483	Asdang Park	Nakhon Rachasima/North-Eastern
140	TMMI250	I69	*Scedosporium apiospermum*	MG204484	Asdang Park	Nakhon Rachasima/North-Eastern

141	TMMI251	I610	*Scedosporium apiospermum*	MG204485	Asdang Park	Nakhon Rachasima/North-Eastern
142	TMMI252	I611	*Scedosporium apiospermum*	MG204486	Asdang Park	Nakhon Rachasima/North-Eastern
143	TMMI253	I612	*Scedosporium apiospermum*	MG204487	Asdang Park	Nakhon Rachasima/North-Eastern
144	TMMI254	I614	*Scedosporium apiospermum*	MG204488	Asdang Park	Nakhon Rachasima/North-Eastern
145	TMMI255	I618	*Scedosporium apiospermum*	MG204489	Asdang Park	Nakhon Rachasima/North-Eastern
146	TMMI256	I620	*Scedosporium apiospermum*	MG204490	Asdang Park	Nakhon Rachasima/North-Eastern
147	TMMI257	I622	*Scedosporium apiospermum*	MG204491	Asdang Park	Nakhon Rachasima/North-Eastern
148	TMMI258	I625	*Scedosporium apiospermum*	MG204492	Asdang Park	Nakhon Rachasima/North-Eastern
149	TMMI259	I627	*Scedosporium apiospermum*	MG204493	Asdang Park	Nakhon Rachasima/North-Eastern
150	TMMI260	J21	*Scedosporium apiospermum*	MG204494	Nongkae Chang Park	Nakhon Rachasima/North-Eastern
151	TMMI261	J23	*Scedosporium apiospermum*	MG204495	Nongkae Chang Park	Nakhon Rachasima/North-Eastern
152	TMMI262	J24	*Scedosporium apiospermum*	MG204496	Nongkae Chang Park	Nakhon Rachasima/North-Eastern
153	TMMI263	J41	*Scedosporium apiospermum*	MG204497	Nongkae Chang Park	Nakhon Rachasima/North-Eastern
154	TMMI264	J44	*Scedosporium apiospermum*	MG204498	Nongkae Chang Park	Nakhon Rachasima/North-Eastern
155	TMMI265	J47	*Scedosporium apiospermum*	MG204499	Nongkae Chang Park	Nakhon Rachasima/North-Eastern
156	TMMI266	J51	*Scedosporium apiospermum*	MG204500	Nongkae Chang Park	Nakhon Rachasima/North-Eastern
157	TMMI267	J72	*Scedosporium apiospermum*	MG204501	Nongkae Chang Park	Nakhon Rachasima/North-Eastern
158	TMMI268	J75	*Scedosporium apiospermum*	MG204502	Nongkae Chang Park	Nakhon Rachasima/North-Eastern
159	TMMI269	J81	*Scedosporium apiospermum*	MG204503	Nongkae Chang Park	Nakhon Rachasima/North-Eastern
160	TMMI270	J83	*Scedosporium apiospermum*	MG204504	Nongkae Chang Park	Nakhon Rachasima/North-Eastern
161	TMMI271	J84	*Scedosporium apiospermum*	MG204505	Nongkae Chang Park	Nakhon Rachasima/North-Eastern
162	TMMI272	J86	*Scedosporium apiospermum*	MG204506	Nongkae Chang Park	Nakhon Rachasima/North-Eastern
163	TMMI273	J104	*Scedosporium apiospermum*	MG204507	Nongkae Chang Park	Nakhon Rachasima/North-Eastern
164	TMMI274	J105	*Scedosporium apiospermum*	MG204508	Nongkae Chang Park	Nakhon Rachasima/North-Eastern
165	TMMI275	J108	*Scedosporium* species	MG204345	Nongkae Chang Park	Nakhon Rachasima/North-Eastern
166	TMMI276	L21	*Scedosporium apiospermum*	MG204509	Bueng Phraram Public Park	Pra Nakhobn Sri Ayuthaya/Central
167	TMMI277	L27	*Scedosporium apiospermum*	MG204510	Bueng Phraram Public Park	Pra Nakhobn Sri Ayuthaya/Central
168	TMMI278	L41	*Scedosporium apiospermum*	MG204511	Bueng Phraram Public Park	Pra Nakhobn Sri Ayuthaya/Central

169	TMMI279	L42	*Scedosporium apiospermum*	MG204512	Bueng Phraram Public Park	Pra Nakhobn Sri Ayuthaya/Central
170	TMMI280	L43	*Scedosporium apiospermum*	MG204513	Bueng Phraram Public Park	Pra Nakhobn Sri Ayuthaya/Central
171	TMMI281	L44	*Scedosporium apiospermum*	MG204514	Bueng Phraram Public Park	Pra Nakhobn Sri Ayuthaya/Central
172	TMMI282	L45	*Scedosporium apiospermum*	MG204515	Bueng Phraram Public Park	Pra Nakhobn Sri Ayuthaya/Central
173	TMMI283	L46	*Scedosporium apiospermum*	MG204516	Bueng Phraram Public Park	Pra Nakhobn Sri Ayuthaya/Central
174	TMMI284	L47	*Scedosporium apiospermum*	MG204517	Bueng Phraram Public Park	Pra Nakhobn Sri Ayuthaya/Central
175	TMMI285	L48	*Scedosporium apiospermum*	MG204518	Bueng Phraram Public Park	Pra Nakhobn Sri Ayuthaya/Central
176	TMMI286	L49	*Scedosporium apiospermum*	MG204519	Bueng Phraram Public Park	Pra Nakhobn Sri Ayuthaya/Central
177	TMMI287	L410	*Scedosporium apiospermum*	MG204520	Bueng Phraram Public Park	Pra Nakhobn Sri Ayuthaya/Central
178	TMMI288	L411	*Scedosporium apiospermum*	MG204521	Bueng Phraram Public Park	Pra Nakhobn Sri Ayuthaya/Central
179	TMMI289	L412	*Scedosporium apiospermum*	MG204522	Bueng Phraram Public Park	Pra Nakhobn Sri Ayuthaya/Central
180	TMMI290	L413	*Scedosporium apiospermum*	MG204523	Bueng Phraram Public Park	Pra Nakhobn Sri Ayuthaya/Central
181	TMMI291	L414	*Scedosporium apiospermum*	MG204524	Bueng Phraram Public Park	Pra Nakhobn Sri Ayuthaya/Central
182	TMMI292	L415	*Scedosporium apiospermum*	MG204525	Bueng Phraram Public Park	Pra Nakhobn Sri Ayuthaya/Central
183	TMMI293	L416	*Scedosporium* species	MG204526	Bueng Phraram Public Park	Pra Nakhobn Sri Ayuthaya/Central
184	TMMI294	L417	*Scedosporium apiospermum*	MG204527	Bueng Phraram Public Park	Pra Nakhobn Sri Ayuthaya/Central

185	TMMI295	L418	*Scedosporium apiospermum*	MG204528	Bueng Phraram Public Park	Pra Nakhobn Sri Ayuthaya/Central
186	TMMI296	L419	*Scedosporium apiospermum*	MG204529	Bueng Phraram Public Park	Pra Nakhobn Sri Ayuthaya/Central
187	TMMI297	L81	*Scedosporium apiospermum*	MG204530	Bueng Phraram Public Park	Pra Nakhobn Sri Ayuthaya/Central
188	TMMI298	L82	*Scedosporium apiospermum*	MG204531	Bueng Phraram Public Park	Pra Nakhobn Sri Ayuthaya/Central
189	TMMI299	Q41	*Scedosporium apiospermum*	MG204532	Municipal Public Park	Prachuap Khiri Khan/Western
190	TMMI300	Q42	*Scedosporium apiospermum*	MG204533	Municipal Public Park	Prachuap Khiri Khan/Western
191	TMMI301	X21	*Scedosporium apiospermum*	MG204534	Chaloem Phrakiat Health Park	Samut songkhram/Central
