# Supplementary material for: Distribution of Scedosporium species in soil from areas with high human population density and tourist popularity in six geographic regions in Thailand
Source: PLoS One. 2019 Jan 23;14(1):e0210942. doi: 10.1371/journal.pone.0210942 (PMC6343921; doi:10.1371/journal.pone.0210942)
Supplement: S2 Table — (PDF) [file pone.0210942.s002.pdf]

S2 Table. Sequences of the reference strains (download from GenBank).

**Reference strains**	**GenBank number**
S. aurantiacum strain CBS 117426	KT008435
S. aurantiacum strain CBS 117414	KT008436
S. aurantiacum strain CBS 103.44	KT008437
S. angusta strain CBS 108.54	KT008442
S. angusta strain CBS 106.53	KT008443
S. minutisporum strain CBS 116595	KT008439
S. minutisporum strain CBS 100396	KT008440
S. cereisporum	KJ599659
S. apiospermum strain CBS 116899	KT008473
S. apiospermum strain CBS 329.93	KT008477
S. apiospermum strain CBS 117411	KT008484
S. boydii strain CBS 117404	KT008453
S. boydii strain CBS 115.59	KT008454
S. boydii strain CBS 117390	KT008465
S. dehoogii strain CBS 499.90	KT008497
S. dehoogii strain CBS 117393	KT008495
S. dehoogii strain CBS 101721	KT008492
L. prolificans strain FMR 7294	AJ889591
P. africana strain CBS 311.72	AJ889603
S. apiospermum strain TMMI001	KY123063
S. apiospermum strain TMMI011	KY123071
S. apiospermum strain TMMI015	KY123072
S. apiospermum strain TMMI016	KY123073
S. apiospermum strain TMMI031	KY123081
S. apiospermum strain TMMI033	KY123034
S. apiospermum strain TMMI043	KY123090
S. apiospermum strain TMMI044	KY123043
S. apiospermum strain TMMI054	KY123099
S. apiospermum strain TMMI071	KY123109
S. apiospermum strain TMMI072	KY123110
